# Supplementary material for: The selective PGI2 receptor agonist selexipag ameliorates Sugen 5416/hypoxia-induced pulmonary arterial hypertension in rats
Source: PLoS One. 2020 Oct 15;15(10):e0240692. doi: 10.1371/journal.pone.0240692 (PMC7561119; doi:10.1371/journal.pone.0240692)
Supplement: S1 File — (DOCX) [file pone.0240692.s001.docx]

Supplemental Materials and Methods

Normal human lung fibroblasts (LONZA Ltd., Basel, Switzerland) were seeded at 5×10^3^ cells/well in 96-well plates and cultured for 24 h. Cells were growth-arrested for 24 h and then incubated for 48 h with or without 10 ng/mL tumor growth factor (TGF) β and MRE-269. The concentration of procollagen type 1 C-peptide in the media was measured by ELISA (Takara Bio Inc., Shiga, Japan) and normalized by it of non-stimulated group.
