# Supplementary figures and images for: The selective PGI2 receptor agonist selexipag ameliorates Sugen 5416/hypoxia-induced pulmonary arterial hypertension in rats
Source: PLoS One. 2020 Oct 15;15(10):e0240692. doi: 10.1371/journal.pone.0240692 (PMC7561119; doi:10.1371/journal.pone.0240692)

## Slide 1
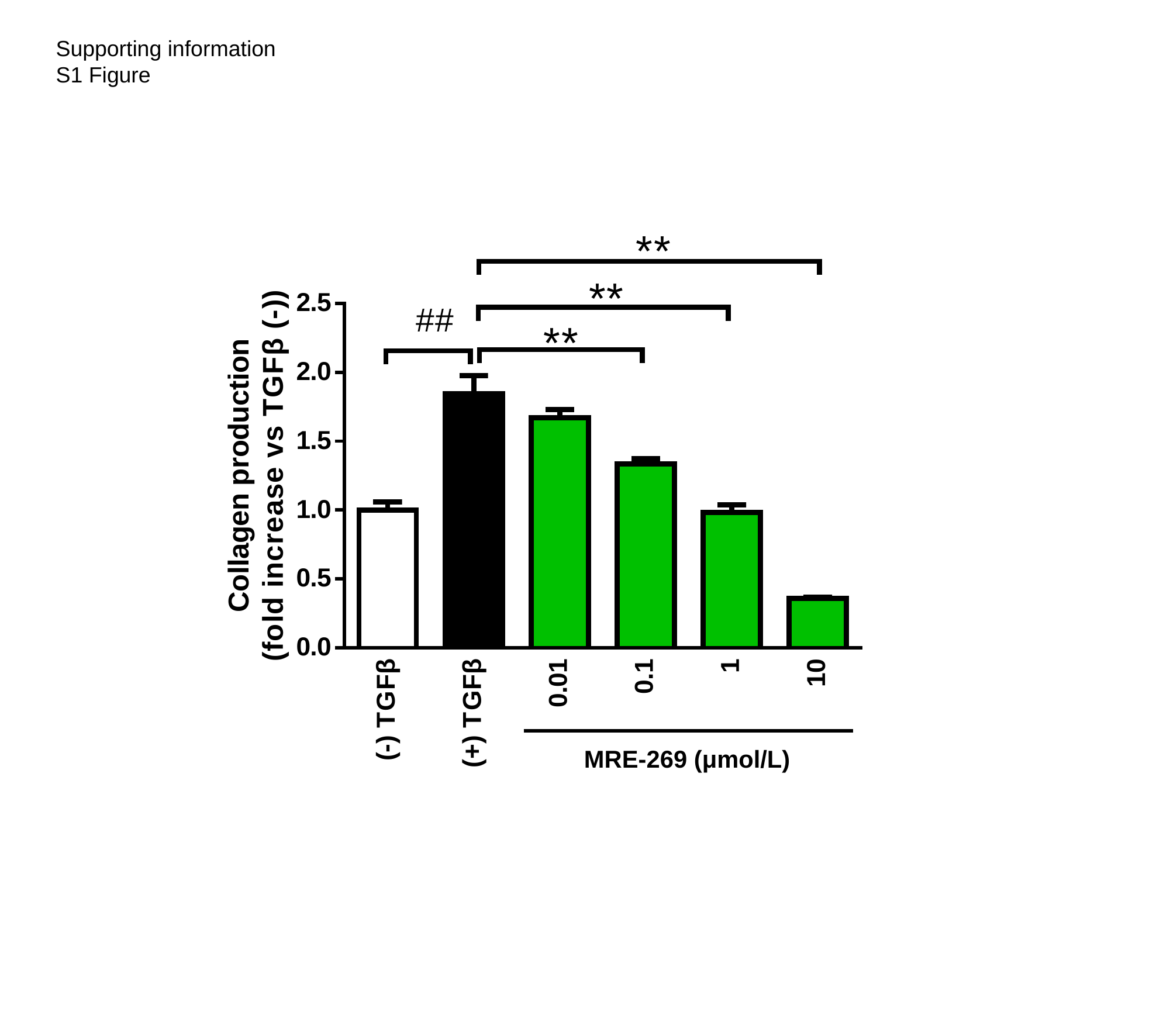

Supporting information
S1 Figure
MRE-269 (μmol/L)

Supplement: S1 Fig — Statistical analyses were performed using Student’s t- test followed by Dunnett’s test. ##P<0.01 vs. TGFβ (-) group by Student’s t-test, **P<0.01 vs. TGFβ (+) group by Dunnett’s test. Values are means ± S.E.M. N = 4 per group. (PPTX) [file pone.0240692.s002.pptx]
